# Supplementary material for: Distinct nonlinear spectrotemporal integration in primary and secondary auditory cortices
Source: Sci Rep. 2023 May 11;13:7658. doi: 10.1038/s41598-023-34731-6 (PMC10175507; doi:10.1038/s41598-023-34731-6)
Supplement: Supplementary file 1 — Supplementary Figures. [file 41598_2023_34731_MOESM1_ESM.docx]

**Supplementary Figure**

**
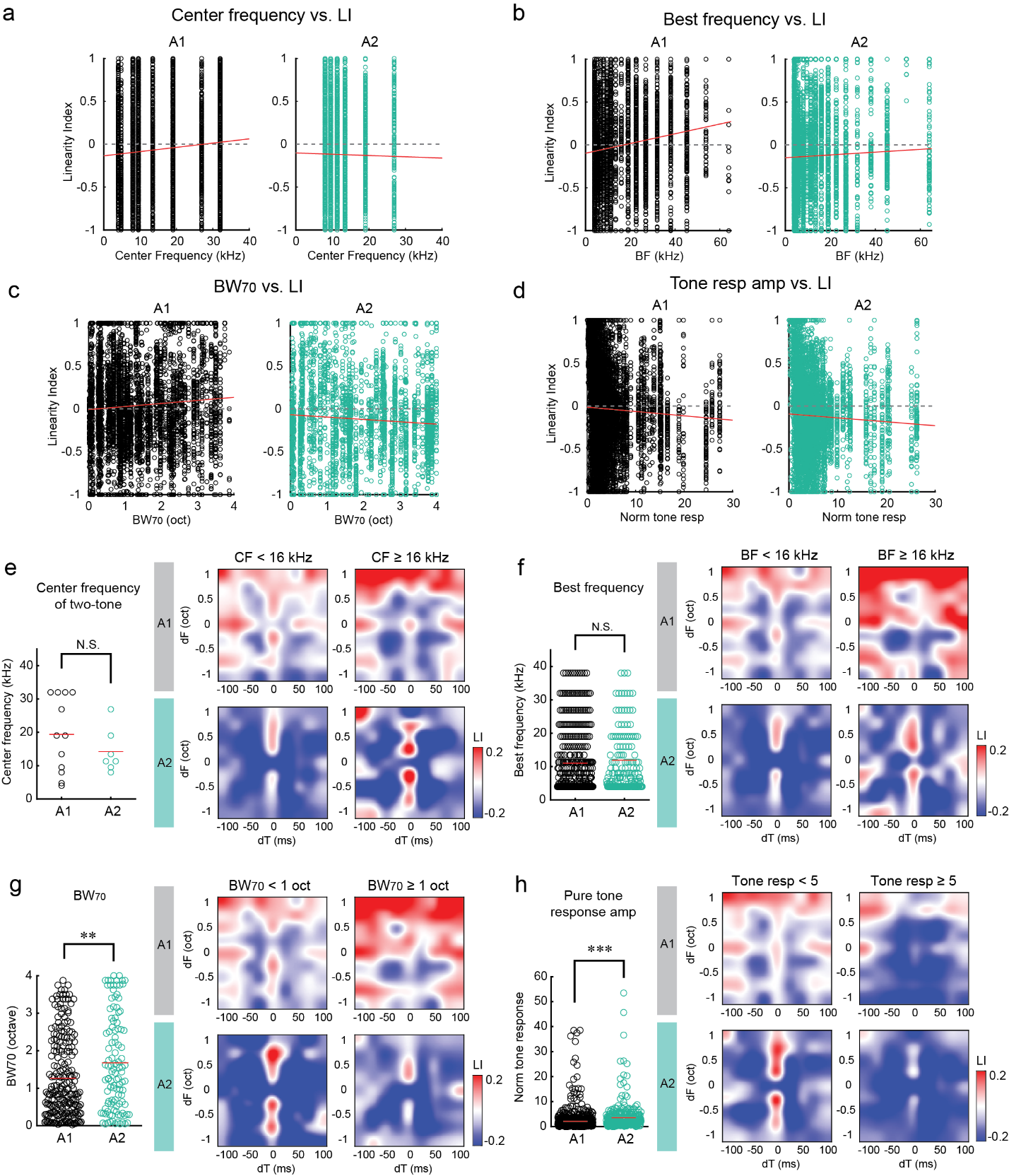
**

**Supplementary Figure 1. Coincidence preference in A2 is not explained by cellular pure tone response properties. (a)** Scatter plots showing the distribution of linearity index against the center frequency of two-tone stimuli in A1 (left) and A2 (right). A1: n = 11809 cell-dF-dT pairs, R = 0.0965; A2: n = 8299, R = -0.0143. Red lines, linear regression. **(b)** Scatter plots showing the distribution of linearity index against individual cells’ best frequency to pure tones. A1: n = 7920 cell-dF-dT pairs, R = 0.1217; A2: n = 4813, R = 0.0368. The dataset includes only cells that showed significant responses to pure tones during the tonal receptive field measurement in separate imaging sessions. **(c)** Scatter plots showing the distribution of linearity index against individual cells’ pure tone response bandwidth at 70 dB SPL (BW_70_). A1: n = 4740 cell-dF-dT pairs, R = 0.0688; A2: n = 3328, R = -0.0710. The dataset includes only cells that showed significant responses to 70 dB SPL pure tones during the tonal receptive field measurement in separate imaging sessions. **(d)** Scatter plots showing the distribution of linearity index against individual cells’ response amplitudes to component tones. A1: n = 11800 cell-dF-dT pairs, R = -0.0588; A2: n = 8295, R = -0.0775. **(e)** Left, scatter plot showing the distribution of center frequency chosen for two-tone stimuli in individual mice. A1: n = 12, A2: n = 7 mice. p = 0.4182. Red lines are mean. Right, spectrotemporal interaction maps in A1 and A2, separated into experiments with center frequency smaller (left) and larger (right) than 16 kHz. **(f)** Same as **(e)**, but for cells with best frequency smaller (left) and larger (right) than 16 kHz. A1: n = 1234, A2: n = 435 cells. p = 0.0615. **(g)** Same as **(e)**, but for cells with BW_70_ smaller (left) and larger (right) than one octave. **p = 0.0049. **(h)** Same as **(e)**, but for cells with normalized pure tone responses smaller (left) and larger (right) than 5. ***p = 5.3×10^-15^.

**
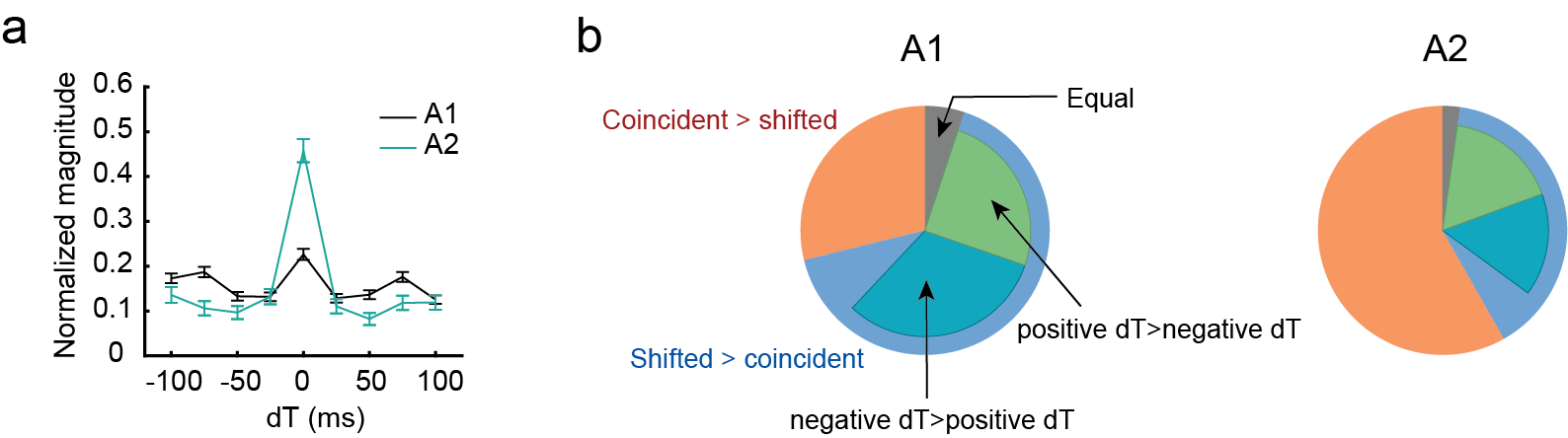
**

**Supplementary Figure 2. Coincidence preference in A2 holds true even if only pure tone-nonresponsive neurons are analyzed. (a)** Summary data comparing normalized response magnitudes in A1 and A2, using only pure tone-nonresponsive but two tone-responsive cells. A1: n = 492, A2: n = 134 cells. Data are mean ± SEM. Note that the linearity index is not meaningful for pure tone-nonresponsive cells. **(b)** A1 and A2 pure tone-nonresponsive neurons classified by their preference for two-tone timings. The fraction of neurons preferring coincident over shifted stimuli was significantly higher in A2 than A1 (A1: 28.9%, A2: 58.2%, Chi-square test, p = 2.82×10^-10^).

**
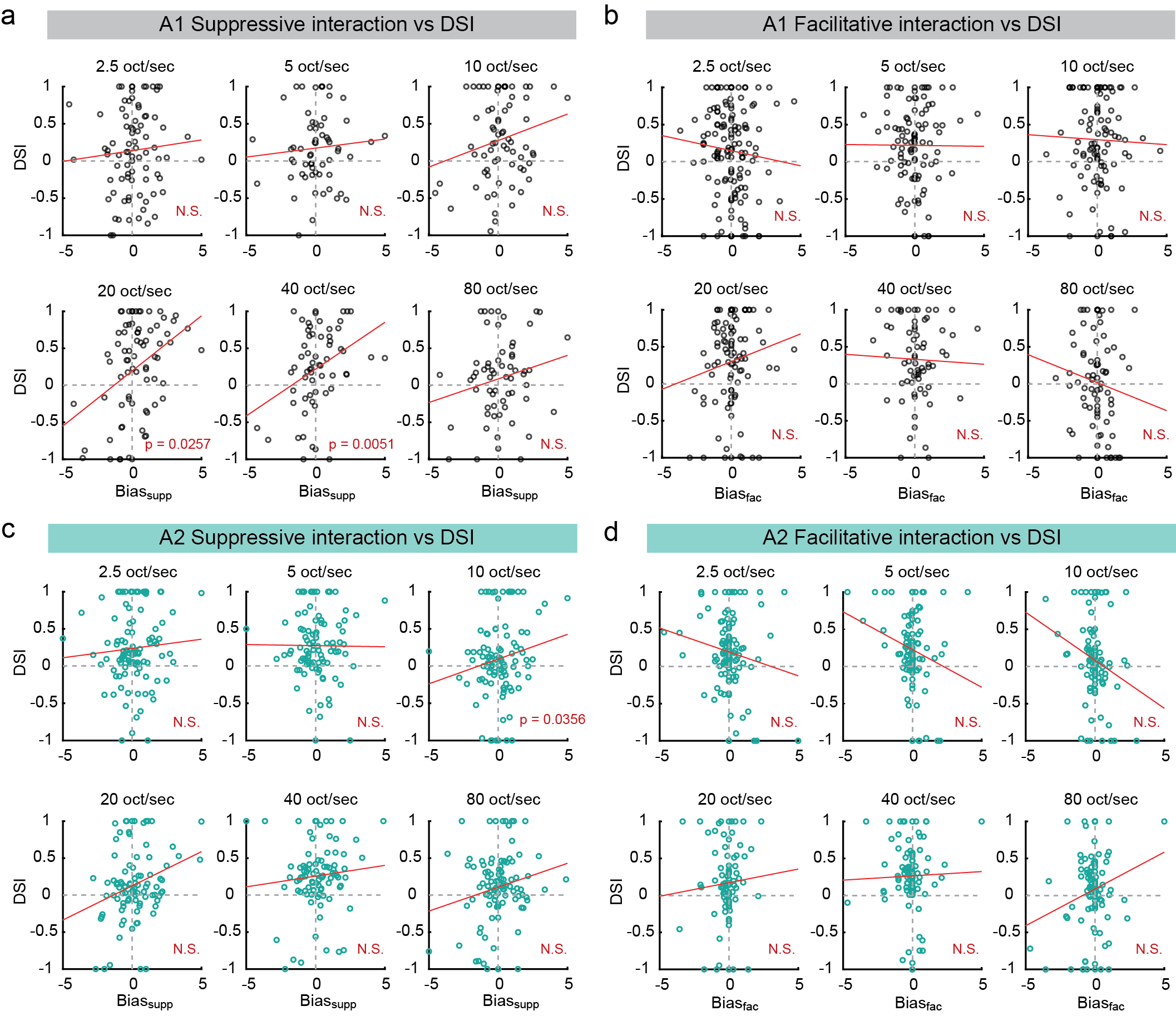
**

**Supplementary Figure 3. Correlation between suppressive nonlinearity bias and direction selectivity is driven by middle-range FM rates.** **(a)** DSI of A1 neurons around middle FM rates (20-40 oct/sec) has a strong correlation with linearity index bias for suppressive interactions (Bias_supp_). Two-sided t test. p values are adjusted for multiple comparisons with Bonferroni Correction. **(b)** There is no correlation for facilitative interactions (Bias_fac_) in A1. **(c-d)** Same as (a) and (b) but for A2 neurons. Red lines, regression curves. A1: n = 220 cells, A2: n = 171 cells responsive to both FM sweeps and two tones.
